# Music Listening Habits and Auditory Functions in Medical College Students

**DOI:** 10.31662/jmaj.2025-0016

**Published:** 2025-05-30

**Authors:** Tomoyasu Ishida, Jun Suzuki, Takeshi Sato, Tetsuya Oishi, Yohei Honkura, Ryoukichi Ikeda, Yukio Katori

**Affiliations:** 1 Department of Otolaryngology, Head and Neck Surgery, Tohoku University Graduate School of Medicine, Sendai, Japan; 2 Department of Otolaryngology, Head and Neck Surgery, Iwate Medical University School of Medicine, Yahaba, Japan

**Keywords:** music, hearing loss, college students, personal listening devices, extended high-frequency audiometry, loudness discomfort level

## Abstract

**Introduction:**

Early detection of hearing impairment in young adults and raising social awareness of preventive measures are essential to mitigate the negative impact on individuals’ quality of life and reduce the societal costs associated with hearing impairment. This study investigated the music listening habits of medical college students using audiological function tests to assess their impact on young adults’ hearing functions.

**Methods:**

We recruited 77 healthy volunteers who underwent a comprehensive assessment, including a questionnaire on music listening habits, pure-tone audiometry (PTA), tympanometry, extended high-frequency (EHF) audiometry, and loudness discomfort level (LDL) tests. Furthermore, we measured preferred music listening levels (PMLs). The association between music listening habits and PMLs and the results of various auditory function tests were examined.

**Results:**

Among the 77 participants, 60 were men, and 17 were women, with a median age of 23 years. All participants exhibited a mean hearing threshold of less than 25 dB HL on PTA. Sixty-six (85.7%) participants used earphones/headphones daily, with a median usage duration of 2.0 hours. The correlations between the duration of earphones/headphones usage, EHF thresholds, and LDLs were not significantly different. The correlation between the duration of earphones/headphones usage and PMLs was weakly positive (r = 0.2772). A stronger positive correlation (r = 0.4625) was observed when focusing on 23 participants with high LDL values.

**Conclusions:**

The positive correlation between the duration of earphones/headphones usage and PMLs suggested that inappropriate music listening habits might synergistically act as risk factors for hearing impairment in young adults.

## Introduction

The World Health Organization (WHO) estimates that over 430 million people currently have hearing loss and require global rehabilitation services ^(1)^. WHO also announced that more than 1 billion young people worldwide are potentially at risk of permanent hearing loss due to unsafe listening practices, such as prolonged and excessive exposure to loud sounds from personal listening devices (PLDs), such as smartphones, and attendance at loud music venues ^(2)^, and these risks are supported by a recent systematic review and meta-analysis ^(3)^. Hearing loss at 4,000 Hz in young people has been reported in Japan over the past 20 years, supporting the problem raised by these backgrounds ^(4)^. Hearing loss hinders communication, reduces the quality of daily life, and contributes to social isolation, depression, and dementia ^(5)^. Therefore, early detection of hearing loss in younger generations and preventive measures against hearing loss are essential to mitigate its negative impact on individuals’ quality of life and reduce the societal costs associated with hearing impairment.

Among noisy recreational activities, using PLDs with earphones/headphones is noteworthy because the frequency of earphones/headphones usage increases to avoid disturbing others. The output volume of most PLDs exceeds the presumed safe volume (80 dB sound pressure level), potentially causing noise-induced hearing loss ^(6), (7)^. Moreover, the unsafe use of PLDs causes headaches, tinnitus, and impaired concentration ^(8), (9)^. The preferred music listening level (PML) significantly increases when using PLDs in noisy circumstances ^(10)^, and the use of PLDs at volume levels above the 100 dB hearing level (HL) is a significant risk factor for hearing impairment ^(11)^. Noise exposure is an established risk factor for sensorineural hearing loss ^(12)^, and acoustic overexposure caused by PLD use can permanently damage sensory cells and other structures, resulting in permanent hearing loss. Animal studies have shown that noise exposure that only induces transient threshold shifts in hearing can cause a permanent reduction in inner hair cell synapses, leading to early-onset age-related hearing loss and tinnitus ^(12)^. This phenomenon, known as “hidden hearing loss” (HHL) ^(13)^, may also occur in humans due to acoustic overexposure from PLD use.

Scattered reports have investigated the risk of hearing impairment among young people, such as college students, concerning their use of PLDs. However, both supportive (i.e., harmful) ^(14), (15), (16)^ and negative (i.e., not harmful) reports ^(17)^ regarding whether unsafe listening habits with PLDs pose an apparent risk factor for hearing loss exist, and a definite conclusion has not been reached. Moreover, the effects of PLD usage on detailed auditory functions, such as extended high-frequency (EHF) hearing thresholds, loudness discomfort level (LDL), and word-recognition scores of speech-in-noise (SIN) audiometry, remain unclear.

This study aimed to collect information on the music listening habits of college students as a basis for awareness-raising activities and compare how differences in music listening habits affect the results of various hearing tests, such as EHF audiometry, LDL, and SIN audiometry.

## Materials and Methods

### Participants

A cross-sectional study was performed among students at Tohoku University School of Medicine between May 2022 and October 2023. Participants were recruited if they met the following criteria: healthy volunteers of medical college students aged ≥20 and <30 years, no history of sensorineural hearing loss and otological surgery, no acute upper airway infections and otitis media, and exhibiting a type-A tympanogram. Informed consent was obtained from all the participants, and their records and information were anonymized before analysis. This study was approved by the Ethics Committee of Tohoku University Graduate School of Medicine (approval number 2020-1-211) and conducted following the guidelines of the Declaration of Helsinki.

### Questionnaires about music listening habits

The following items were investigated: age, sex, past medical history including ear diseases, family history of hearing loss, presence of aural symptoms (hearing loss, tinnitus, ear fullness, etc.), frequency of concert attendance, playing musical instruments, total daily earphones/headphones usage time, noise-canceling function of their earphones/headphones, listening location (indoors or outdoors), and awareness of loud volume during earphones/headphones usage.

### Hearing tests

Pure-tone audiometry (PTA) and LDL tests (125-8,000 Hz) with air conduction were performed in a soundproof room with a conventional audiometer (AA-H1; RION Co., Ltd., Tokyo, Japan) and headphones (AD-02T; RION Co., Ltd.) using a modified Hughson-Westlake procedure. EHF audiometry (10,000, 12,000, 14,000, and 16,000 Hz) with air conduction was performed using a conventional audiometer (AA-H1) and an HDA 200 audiometric headphone (Sennheiser, Wedemark, Germany). Bone conduction tests were conducted across frequencies ranging from 250-4,000 Hz. Tympanometry was conducted using an impedance audiometer RS-H1 (RION Co., Ltd.). Distortion product otoacoustic emissions (DPOAEs) were measured using an OAE screener ER-60 (RION, Co., Ltd.). The SIN test was performed using an audiometer (AA-H1) and headphones (AD-02T) on 37 participants. Speech intelligibility was assessed for 50 Japanese monosyllables lists presented at 60 dB HL without noise or with background continuous speech noise at 50 dB HL (signal-to-noise ratio [S/N] +10 dB) or 55 dB HL (S/N = +5 dB). Word-recognition scores were calculated as percentages of correct answers. Because the SIN test was added as an examination item during this study, it was not performed on all cases. All hearing tests were performed by a medical technologist or a speech-language-hearing therapist. The hearing test values were shown as the average of the left and right values.

### Evaluation of the PML

Participants listened to specific rock music (Linkin Park, “Numb”) using an iPod touch (7th generation) and EarPods (Apple, Cupertino, CA, USA) in a soundproof room. They were instructed to set the volume level to their preference and typical listening level, which was then verified using the Healthcare App (Apple).

### Statistical analysis

All statistical analyses were performed using Prism 9 (GraphPad Software, San Diego, CA, USA) for the Mann-Whitney U, Fisher’s exact, and Spearman’s correlation tests. Data were presented as median and interquartile ranges. Statistical significance was set at p < 0.05.

## Results

### Participant characteristics and PMLs

Table 1 shows the participant characteristics. In total, 77 healthy volunteers (60 men and 17 women) with a median age of 23 years (range: 22-24 years) were included in this study. Fifteen (19.5%) participants presented with aural symptoms. Table 2 shows the results of questionnaires on music listening habits and PMLs while using earphones/headphones. Sixty-six (85.7%) participants used earphones/headphones daily for a median of 2.0 (1.0-3.0) hours per day. Approximately 60% of the participants (39/66) used earphones/headphones with noise-canceling functions. Fourteen (18.2%) participants were aware that the volume of their music was loud when they used the earphones/headphones. The median PML in the soundproof box was 52.3 (45.6-57.0) dB HL. No significant sex-based differences were observed concerning aural symptoms, concert attendance, playing musical instruments, music listening habits, and PMLs.

**Table 1.** Participant Characteristics.

		All, n=77	Male, n=60	Female, n=17	p value
Age (years) (median [IQR])		23 [22-24]	23 [22-24]	22 [22-23]	0.139
Past ear diseases	Yes (otitis media)	17 (22.1%)	13 (21.7%)	4 (23.5%)	>0.999
	No	60 (77.9%)	47 (78.3%)	13 (76.5%)	
Aural symptoms	Yes	15 (19.5%)	13 (21.7%)	2 (11.8%)	0.499
	(Ear fullness)	4 (5.2%)	2 (3.3%)	2 (11.8%)	
	(LDs in noise)	6 (7.8%)	6 (10.0%)	0 (0.0%)	
	(Tinnitus)	5 (6.5%)	5 (8.3%)	0 (0.0%)	
	No	62 (80.5%)	47 (78.3%)	15 (88.2%)	

IQR, interquartile range; LDs, listening difficulties.

**Table 2:** Results of Questionnaires of Music Listening Habits and Preferred Music-Listening Levels while Using Earphones/Headphones.

		All, n=77	Male, n=60	Female, n=17	p value
Concert attendance (more than once/month)	Yes	3 (3.9%)	1 (1.7%)	2 (11.8%)	0.121
	No	74 (96.1%)	59 (98.3%)	15 (88.2%)	
Playing musical instruments	Yes	13 (16.9%)	9 (15.0%)	4 (23.5%)	0.467
	No	64 (83.1%)	51 (85.0%)	13 (76.5%)	
Earphones/headphones usage	Yes	66 (85.7%)	53 (88.3%)	13 (76.5%)	0.247
	(Earphones)	45 (58.4%)	35 (58.3%)	10 (58.8%)	
	(Headphones)	7 (9.1%)	6 (10.0%)	1 (5.9%)	
	(Both)	14 (18.2%)	12 (20.0%)	2 (11.8%)	
	No	11 (14.3%)	7 (11.7%)	4 (23.5%)	
Noise-canceling function: users only (n=66)	Yes	39 (59.1%)	28 (52.8%)	11 (84.6%)	0.058
	No	27 (40.9%)	25 (47.2%)	2 (15.4%)	
Outdoor usage of earphones/headphones	Yes	34 (44.2%)	25 (41.7%)	9 (53.0%)	0.4
	No	41 (53.2%)	34 (56.7%)	7 (41.1%)	
	N/A	2 (2.6%)	1 (1.7%)	1 (5.9%)	
Loud volume awareness	Yes	14 (18.2%)	12 (20.0%)	2 (11.8%)	0.723
	No	63 (81.8%)	48 (80.0%)	15 (88.2%)	
Total earphones/headphones usage (hours) (median [IQR])		1.8 [1.0-3.0]	1.0 [0.3-2.0]	1.0 [0.5-3.0]	0.0704
Total earphones/headphones usage (hours): users only (n=66) (median [IQR])		2.0 [1.0-3.0]	2.0 [1.0-3.0]	1.0 [0.8-2.0]	0.166
PML (dB HL) (median [IQR])		52.3 [45.6-57.0]	52.5 [47.0-57.5]	52.0 [42.0-55.5]	0.242

IQR, interquartile range; PML, preferred music-listening level

### Audiological results

We performed various hearing tests to investigate the auditory functions of the participants in detail (Table 3). All participants showed pure-tone averages of 25 dB HL or less, implying normal general hearing functions. Although the difference was slight, the pure-tone averages for women were significantly lower than those for men (p = 0.004). The mean threshold of EHF was under 20 dB HL (5.0 [1.9-11.3] dB HL). The speech recognition score without noise was 98% (97%-98%). No significant differences were found between men and women in the mean thresholds of EHFs, mean LDLs, or word-recognition scores of SIN tests. Collectively, most participants in this study had normal hearing function.

**Table 3:** Hearing Test Results.

		All, n=77	Male, n=60	Female, n=17	p value
Pure-tone average (500-4,000 Hz) (dB HL) (median [IQR])		5.6 [4.4-9.4]	7.2 [5.0-9.5]	5.0 [3.1-5.6]	0.004*
EHF mean (10,000-16,000 Hz) (dB HL) (median [IQR])		5.0 [1.9-11.3]	5.0 [1.7-10.8]	6.3 [2.5-11.9]	0.699
LDL mean (500-4,000 Hz) (dB HL) (median [IQR])		102.5 [96.3-111.3]	103.1 [95.9-111.4]	102.5 [99.4-111.3]	0.968
Word recognition score (%) (n=37: Male, n=30; Female, n=7) (median [IQR])	No noise	98.0 [97.0-98.0]	98.0 [96.3-98.8]	98.0 [97.0-98.0]	0.893
	S/N +10 dB	78.0 [74.0-80.0]	78.0 [73.3-80.0]	79.0 [75.5-80.0]	0.587
	S/N +5 dB	60.0 [54.0-64.0]	58.0 [54.0-64.0]	63.0 [57.5-63.5]	0.537

IQR, interquartile range; HL, hearing level; EHF, extended high-frequency; LDL, loudness discomfort level; S/N, signal-to-noise ratio.

### Comparison by presence or absence of EHF threshold abnormality

**Figure 1.**
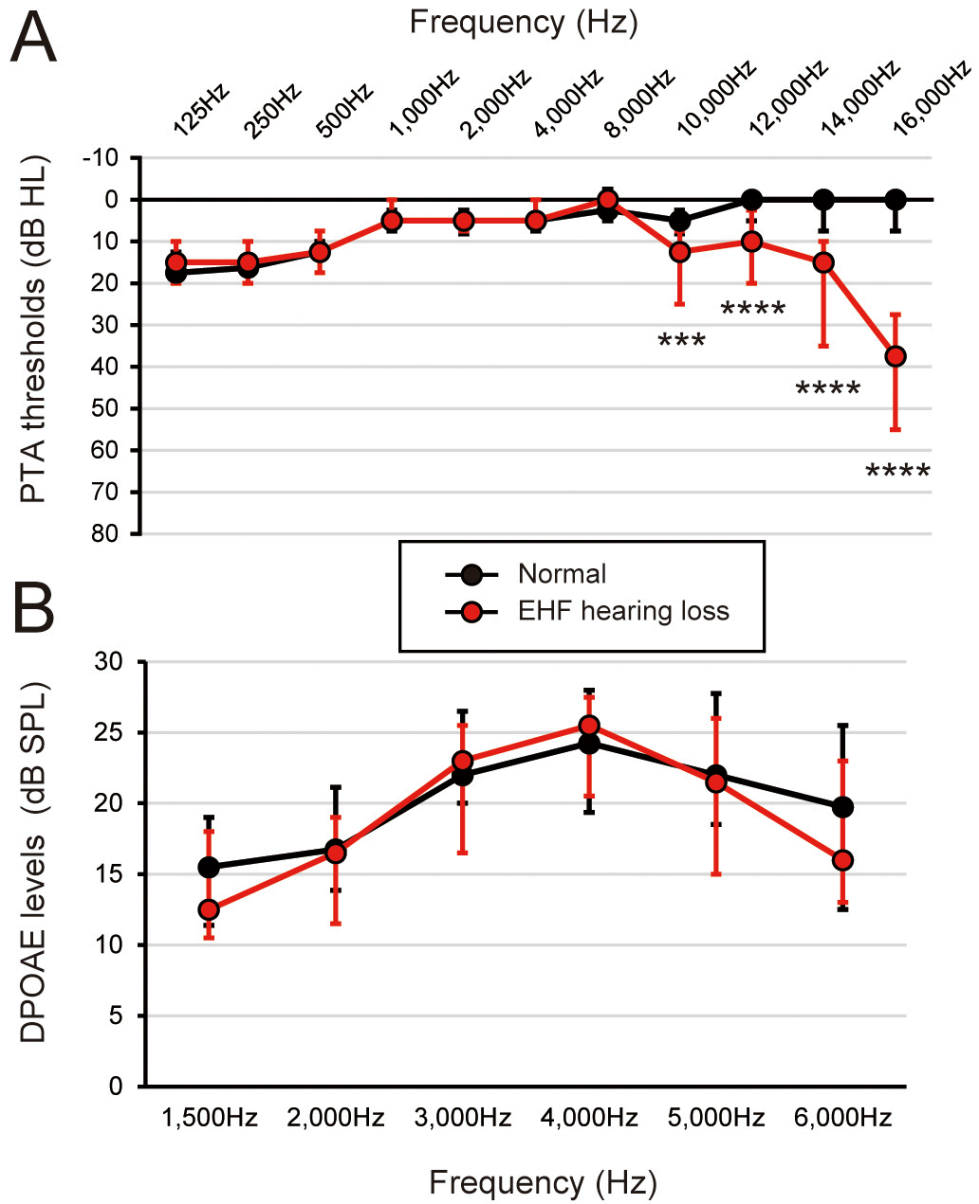
Hearing test results of the normal and extended high-frequency (EHF) hearing loss groups. (A) Thresholds of pure-tone audiometry (PTA). (B) Emission levels (from the noise floor) of the distortion product otoacoustic emissions (DPOAEs). Values are shown as medians, and error bars represent interquartile ranges. Asterisks denote statistical significance (p < 0.05). Significance levels: p < 0.001 (***), p < 0.0001 (****).

To assess the effects of music listening habits on EHF hearing loss, we comprehensively compared various factors and hearing test results between the normal and EHF hearing loss groups, defined as having one or more EHF regions with a threshold of 20 dB HL or more (Table 4). Thresholds of PTA and DPOAEs for each group are shown in Figure 1. No significant differences were observed in the standard PTA frequencies (125-8,000 Hz) and DPOAEs between the two groups. Except for the mean thresholds of EHFs (10,000-16,000 Hz) (3.1 [1.3-6.3] dB HL vs. 20.0 [15.0-23.1] dB HL, p < 0.0001), no significant differences were found between the two groups regarding participant characteristics, playing musical instruments, earphones/headphones usage, PMLs, mean thresholds of PTA, LDLs, and word-recognition scores of SIN tests. These results suggest that no discernible music listening habits were associated with the presence of abnormalities in EHF hearing.

**Table 4:** Comparison by Presence or Absence of Extended High-Frequency Hearing Loss.

		EHF normal, n=60	EHF abnormal, n=17	p value
Age (years) (median [IQR])		23.0 [22.0-23.3]	23.0 [22.0-24.0]	0.312
Sex	Male	48 (80.0%)	12 (70.6%)	0.509
	Female	12 (20.0%)	5 (29.4%)
Playing musical instruments	Yes	8 (13.3%)	5 (29.4%)	0.146
	No	52 (86.7%)	12 (70.6%)
Earphones/headphones usage	Yes	52 (86.7%)	14 (82.3%)	0.699
	(Earphones)	35 (58.3%)	10 (58.8%)
	(Headphones)	5 (8.3%)	2 (11.8%)
	(Both)	12 (20.0%)	2 (11.8%)
	No	8 (13.3%)	3 (17.6%)
Outdoor usage of earphones/headphones	Yes	28 (46.7%)	6 (35.3%)	0.414
	No	30 (50.0%)	11 (64.7%)
	N/A	2 (3.3%)	0 (0.0%)
Loud volume awareness	Yes	12 (20.0%)	2 (11.8%)	0.723
	No	48 (80.0%)	15 (88.2%)
Total earphones/headphones usage (hours) (median [IQR])		1.3 [0.7-3.0]	1.0 [0.3-3.0]	0.606
PML (dB HL) (median [IQR])		52.0 [45.6-57.0]	53.8 [46.8-57.5]	0.74
Pure-tone average (500-4,000 Hz) (dB HL) (median [IQR])		6.3 [4.8-9.4]	5.6 [4.4-8.1]	0.577
EHF mean (10,000-16,000 Hz) (dB HL) (median [IQR])		3.1 [1.3-6.3]	20.0 [15.0-23.1]	<0.0001*
LDL mean (500-4,000 Hz) (dB HL) (median [IQR])		101.3 [94.7-110.6]	109.4 [102.5-111.9]	0.129
Word recognition score (%) (n=37: EHF normal, n=29; EHF abnormal, n=8) (median [IQR])	No noise	98.0 [97.0-98.0]	97.0 [95.8-98.0]	0.192
	S/N +10 dB	77.0 [74.0-80.0]	80.0 [78.8-81.3]	0.102
	S/N +5 dB	60.5 [54.0-65.0]	58.0 [56.3-60.8]	0.494

IQR, interquartile range; LDs, listening difficulties; HL, hearing level; PML, preferred music-listening level; EHF, extended high-frequency; LDL, loudness discomfort level; S/N, signal-to-noise ratio; N/A, not available.

### Comparison by presence or absence of the awareness of loud volume with earphones/headphones

To investigate the effect of loud volume awareness on earphones/headphones, we comprehensively compared various factors and hearing test results based on the presence or absence of awareness of loud volumes when participants usually used earphones/headphones (Table 5). We found no significant differences in participant characteristics and hearing test results, and, as predicted, the PML was significantly higher in participants with loud volume awareness (51.0 [44.0-57.0] dB HL vs. 55.0 [51.0-70.0] dB HL, p = 0.031).

**Table 5:** Comparison by Presence or Absence of Awareness of Loud Volume with Earphones/Headphones.

		Loud volume (-), n=63	Loud volume (+), n=14	p value
Age (years) (median [IQR])		23.0 [22.0-24.0]	23.0 [22.0-23.8]	0.873
Sex	Male	48 (76.2%)	12 (85.7%)	0.753
	Female	15 (23.8%)	2 (14.3%)
Playing musical instruments	Yes	11 (17.4%)	2 (14.3%)	>0.999
	No	52 (82.6%)	12 (85.7%)
Earphones/headphones usage	Yes	52 (82.6%)	14 (100.0%)	0.199
	(Earphones)	35 (55.6%)	10 (71.4%)
	(Headphones)	7 (11.1%)	0 (0.0%)
	(Both)	10 (15.9%)	4 (28.6%)
	No	11 (17.4%)	0 (0.0%)
Outdoor usage of earphones/headphones	Yes	27 (42.9%)	7 (50.0%)	0.771
	No	34 (54.0%)	7 (50.0%)
	N/A	2 (3.2%)	0 (0.0%)
Total earphones/headphones usage (hours) (median [IQR])		1.0 [0-3.0]	2.0 [0.75-3]	0.166
PML (dB HL) (median [IQR])		51.0 [44.0-57.0]	55.0 [51.0-70.0]	0.031*
Pure-tone average (500-4,000 Hz) (dB HL) (median [IQR])		5.0 [4.0-8.0]	8.0 [4.5-10.3]	0.19
EHF mean (10,000-16,000 Hz) (dB HL) (median [IQR])		5.0 [1.0-11.0]	3.0 [1.0-9.5]	0.526
LDL mean (500-4,000 Hz) (dB HL) (median [IQR])		102.0 [95.0-111.0]	105.0 [95.3-111.5]	0.766
Word recognition score (%) (n=37: Loud volume (-), n=30; Loud volume (+), n=7) (median [IQR])	No noise	98.0 [97.0-98.3]	97.0 [96.0-98.0]	0.499
	S/N +10 dB	78.5 [74.0-80.0]	76.0 [73.0-80.0]	0.535
	S/N +5 dB	60.0 [54.0-64.3]	59.0 [53.0-64.0]	0.794

IQR, interquartile range; LDs, listening difficulties; HL, hearing level; PML, preferred music-listening level; EHF, extended high-frequency; LDL, loudness discomfort level; S/N, signal-to-noise ratio; N/A, not available.

### Correlation among the PML, earphones/headphones usage time, EHF thresholds, and uncomfortable listening levels

**Figure 2.**
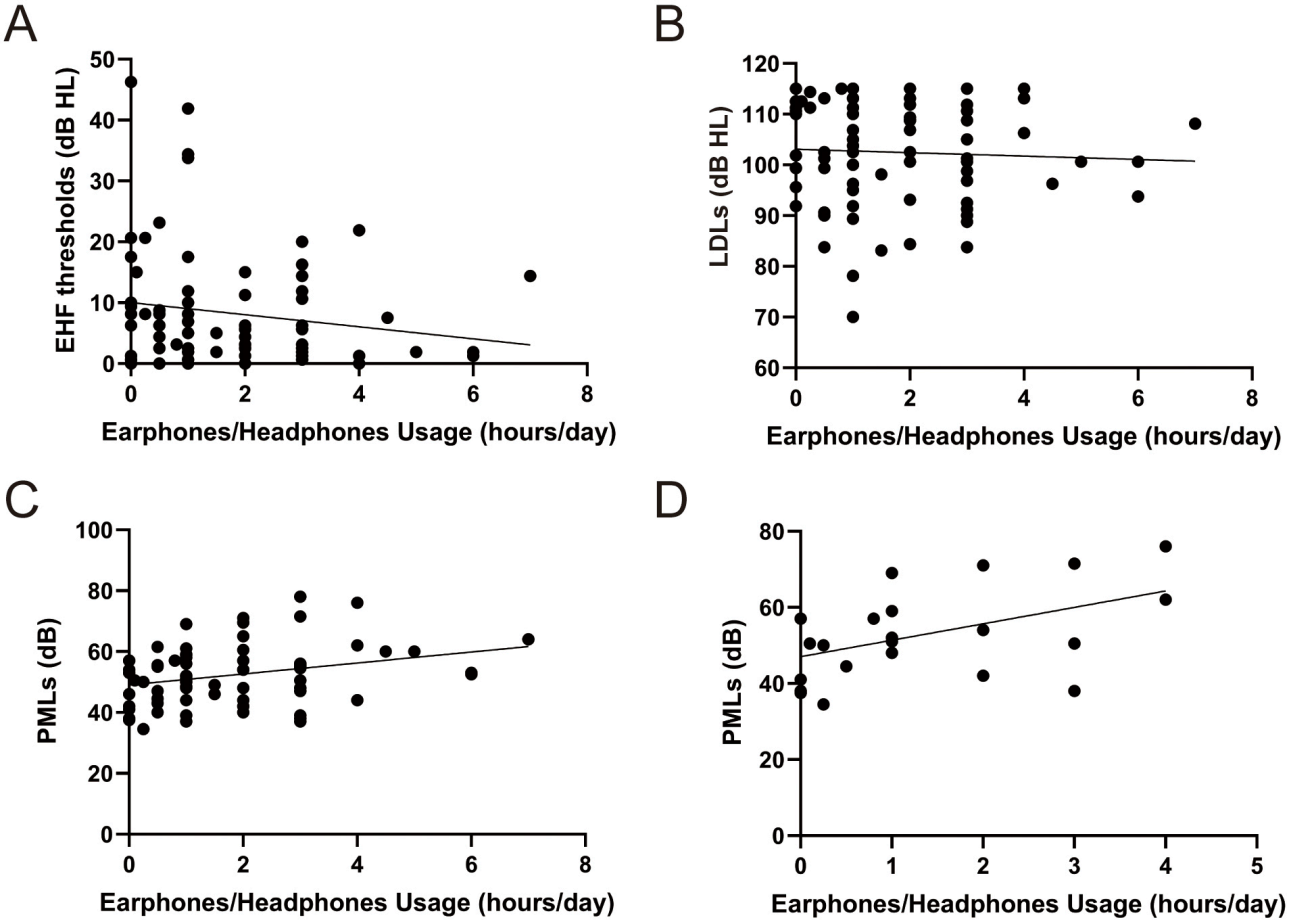
Correlation among earphones/headphones usage time, extended high-frequency (EHF) thresholds, loudness discomfort levels (LDLs), and preferred music listening levels (PMLs). (A) Correlation between earphones/headphones usage time and EHF thresholds. (B) Correlation between earphones/headphones usage time and LDLs. (C) Correlation between earphones/headphones usage time and PMLs (r = 0.277, p = 0.018). (D) Correlation between earphones/headphones usage time and PMLs (r = 0.463, p = 0.026) (n = 23, the participants with LDLs of 110 dB hearing level (HL) or higher).

We performed correlation analyses to assess the relationship between music listening habits and detailed hearing function (Figure 2). No significant correlations were observed between earphones/headphones usage time and EHF thresholds (Figure 2A), earphones/headphones usage time and LDLs (Figure 2B), PMLs and EHF thresholds, and PMLs and EHF thresholds. Interestingly, earphones/headphones usage time and PMLs were significantly positively correlated (r = 0.2772) (Figure 2C), and this correlation became stronger (r = 0.4625) when only participants with high LDLs (110 dB HL or higher) were included (Figure 2D).

## Discussion

In this study, we investigated the association between music listening habits and detailed auditory functions in medical college students. No significant differences were identified between groups with and without EHF hearing loss. We also found no significant differences between the groups with and without awareness of loud music listening with earphones/headphones, except for PMLs. However, we identified a positive correlation between the duration of earphones/headphones usage and PMLs, suggesting that inappropriate music listening habits might act synergistically. Although these results show that earphone/headphone usage within a proper range does not pose an obvious risk of hearing impairment in young adults, they imply that people with risky music listening habits may have another bad habit, suggesting the need for more awareness-raising activities for ear protection in the future.

Evaluation of EHF hearing has been recognized as a helpful method in early predicting hearing impairments in certain conditions, such as age-related hearing loss and cisplatin-induced hearing loss ^(17), (18)^. Furthermore, EHF audiometry is beneficial for detecting populations at high risk of HHL ^(19)^. Past studies have reported worsening EHF thresholds in the habitual music listening group ^(17), (20), (21)^. Longer years of PLD use and higher listening volume have been shown to lead to greater increases in EHF thresholds; however, EHF thresholds are difficult to compare with others because they have large individual differences and no established baseline data for age-specific thresholds ^(20)^. In this study, despite the population being potentially at high risk of acoustic overexposure from earphone/headphone use with subjectively loud volumes, we found no significant differences in EHF thresholds. Conversely, no obvious risk factors were identified when comparing the groups with and without EHF threshold abnormalities (Table 4). Participants in this study, being medical college students, might have exhibited hearing-protective behaviors due to their medical knowledge and health literacy. The frequency of concert attendance, a representative source of leisure noise exposure, was low; the majority had no attendance or attended a few times per year (96.1%, 74/77), and the median PML in a soundproof room was <60 dB HL, even in the loud volume awareness group. These results support previous reports that regular PLD use by university students does not surpass risky levels, i.e., 85 dBA Leq (8 hours) ^(22)^, that the majority of adolescents had listening habits considered safe ^(23)^, and that the music listening levels chosen by student members in the faculty of rehabilitation medicine are not high and of such significant concern ^(10)^. However, young people at exceptionally high risk of acoustic exposure, such as music semi-professionals, were not included in this study. Therefore, future studies should focus on college students at high risk of acoustic exposure, such as brass bands and popular music band members. In this study, we did not conduct investigations into the effects of different earphone/headphone types (closed-type, open-type, etc.) or actual listening volume settings. However, it is possible that these differences may affect hearing in people at high risk of acoustic exposure, so it would be desirable to incorporate this in further research. Additionally, studies have highlighted low education levels and risky behaviors like alcohol consumption as potential risk factors for hearing loss among early adolescents ^(24)^, underscoring the importance of investigating these factors among young people in their 20s.

LDLs are loudness levels at which sounds become uncomfortable and not painful, often used in adjusting hearing aids and assessing hyperacusis ^(25)^. Although the average LDL of normal-hearing individuals is approximately 100 dB HL ^(26)^, the criteria for hyperacusis have not yet been established ^(27)^. Moreover, decreased LDLs have been reported in patients with tinnitus despite having normal hearing ^(28)^. In this study, most participants’ median LDLs were approximately 100 dB HL, consistent with a previous report. Although we found no differences in LDLs regarding EHF abnormality and loud volume awareness, we observed a significant positive correlation between the duration of earphone/headphone usage and PMLs, which became stronger when restricted to participants with LDLs beyond 110 dB HL. These results suggest that measuring LDLs may help screen young people at a higher risk of acoustic overexposure because of their inappropriate music listening habits. Although LDLs have attracted attention as a test for HHL and hyperacusis ^(28)^, they have not been considered a modulating factor for acoustic overexposure in young people. Further investigations focusing on LDLs and music listening habits may help establish an effective prevention strategy for noise-induced hearing loss in young people.

Public interest in hearing loss prevention in the youth has increased following WHO warnings ^(1), (2)^. Notably, many young people lack awareness of hearing loss ^(29), (30)^. Only 8% of young people recognize hearing loss as an important issue ^(31)^, and approximately 75% of university students believe that hearing loss does not occur until old age and that young people are not vulnerable to noise ^(14)^. Although our study did not include a survey of attitudes toward hearing loss, the fact that about half of the participants used noise-canceling earphones/headphones and most participants did not listen to loud music (approximately 55 dB HL) in a quiet environment, compared to the previously reported PML at 70 dB HL ^(32)^, implies that the participants of our study had a high awareness of the problems associated with hearing loss and ear protection. In general, PLD users tend to choose a S/N ratio of 13 dBA ^(7), (32)^. Therefore, the music listening volume may be excessive in noisy environments. Moreover, music listening volume decreases from earphones to headphones and noise-canceling headphones ^(10)^ and music listening in noisy underground environments increases the risk of hearing loss; however, the risk of hearing loss can be avoided by noise-canceling functions ^(33)^. Collectively, the active use of the noise-canceling function of earphones/headphones is recommended to protect youth. Specific recommendations for volume limits during PLD usage have also been announced: up to 90 min/day at up to 80% of the maximum volume ^(34)^ and up to 60 min/day at up to 60% of the maximum volume ^(35)^. Raising awareness about adhering to volume limits while using PLDs is crucial. Additionally, reevaluating the adequacy of setting time and volume limits in the future is essential.

### Limitation

This study had several limitations. Firstly, the study was conducted among medical college students, limiting the generalizability of the findings due to the participants’ high health literacy and educational levels. However, our results reflect the current situation of young Japanese people regarding the use of PLDs. Secondly, the years of PLD usage were not assessed despite evidence suggesting that EHF thresholds worsen with prolonged PLD use ^(20)^. However, given that most participants were aged 22-24 years, significant variations in the duration of PLD usage are unlikely. Thirdly, in this study, the SIN test was administered to only 37 participants, approximately 50% of the total participants, because this test was not included in the original research plan and we started it in the middle of the research. Consequently, detailed analyses related to the SIN tests were not performed. Although there are many previous reports on EHF audiometry and SIN tests, studies examining the association among PML, LDL, and SIN tests are still needed. Therefore, further research development in this area is warranted. Finally, our survey was questionnaire-based, and actual noise exposure status could not be assessed. Developing applications and devices that can measure music listening and noise exposure levels in real time is a challenge for the future.

### Conclusion

The relationship between music listening habits, mainly concerning PLD usage, and various auditory functions among medical college students was investigated. However, no obvious risk factors were identified, possibly because of the high health literacy of the participants in this study. We found a possible synergistic effect on risky music listening habits, such as higher listening volume and prolonged earphones/headphones usage. Further research is warranted to explore this relationship more comprehensively.

## Article Information

### Conflicts of Interest

None

### Acknowledgement

We thank Editage (www.editage.jp) for the English language editing.

### Author Contributions

Conceptualization: Jun Suzuki, Tetsuya Oishi, Takeshi Sato

Data collection and visualization: Tomoyasu Ishida, Takeshi Sato, Jun Suzuki

Writing - original draft: Jun Suzuki, Tomoyasu Ishida

Writing - review and editing: Ryoukichi Ikeda, Yohei Honkura, Yukio Katori

### Approval by Institutional Review Board (IRB)

This study was approved by the Ethical Research Committee of the Tohoku University Graduate School of Medicine (number 2020-1-211).

### Data Availability Statement

Data are available upon reasonable request.
